# Microbial electricity-driven anaerobic phenol degradation in bioelectrochemical systems

**DOI:** 10.1016/j.ese.2023.100307

**Published:** 2023-07-26

**Authors:** Shixiang Dai, Falk Harnisch, Micjel Chávez Morejón, Nina Sophie Keller, Benjamin Korth, Carsten Vogt

**Affiliations:** a Department of Environmental Microbiology, Helmholtz Centre for Environmental Research GmbH - UFZ, Leipzig, Germany; b Department of Isotope Biogeochemistry, Helmholtz Centre for Environmental Research GmbH - UFZ, Leipzig, Germany

**Keywords:** Microbial electroremediation, *Geobacter*, Extracellular electron transfer, Anaerobic phenol degradation, Microbial syntrophy

## Abstract

Microbial electrochemical technologies have been extensively employed for phenol removal. Yet, previous research has yielded inconsistent results, leaving uncertainties regarding the feasibility of phenol degradation under strictly anaerobic conditions using anodes as sole terminal electron acceptors. In this study, we employed high-performance liquid chromatography and gas chromatography-mass spectrometry to investigate the anaerobic phenol degradation pathway. Our findings provide robust evidence for the purely anaerobic degradation of phenol, as we identified benzoic acid, 4-hydroxybenzoic acid, glutaric acid, and other metabolites of this pathway. Notably, no typical intermediates of the aerobic phenol degradation pathway were detected. One-chamber reactors (+0.4 V vs. SHE) exhibited a phenol removal rate of 3.5 ± 0.2 mg L^−1^ d^−1^, while two-chamber reactors showed 3.6 ± 0.1 and 2.6 ± 0.9 mg L^−1^ d^−1^ at anode potentials of +0.4 and + 0.2 V, respectively. Our results also suggest that the reactor configuration certainly influenced the microbial community, presumably leading to different ratios of phenol consumers and microorganisms feeding on degradation products.

## Highlights

• Strong evidence for anaerobic phenol degradation using anode as sole terminal electron acceptors.
• Identification of key metabolites, such as 4-hydroxybenzoic acid and glutaric acid.
• Reactor configuration had a minor impact on phenol degradation.
• *Geobacter* and *Arcobacter* were most abundant in two-chamber reactors.
• *Syntrophorhabdus* seemingly has a central role in mutualistic metabolisms.

## Graphical abstract

**Figure:**
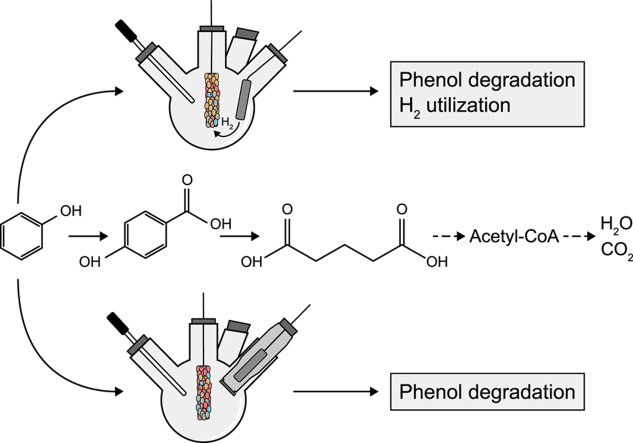

## 1 Introduction

Phenol is a common pollutant discharged by many industries, such as chemical, petrochemical, pharmaceutical, and pesticide sectors [1]. Given its harmful effects on human health, including toxicity, mutagenicity, and carcinogenicity, and its ability to extensively diffuse and persist in the environment, effective treatment methods for phenol are crucial in order to mitigate its associated adverse impacts [1,2].

Various methods are employed for the removal from wastewater including abiotic, such as adsorption and advanced oxidation, and biological methods, including aerobic and anaerobic microbial phenol degradation [3,4]. Among these methods, microbial degradation is often favored due to its cost-efficiency and environmental-friendly properties [4]. Moreover, because of the widespread occurrence of phenol in the environment, phenol-degrading microorganisms are ubiquitous in numerous habitats [5]. However, one apparent bottleneck of microbial phenol degradation is the need for a sufficient supply of electron acceptors, such as oxygen under aerobic conditions or nitrate, sulfate, Fe(III), and Mn(IV) under anaerobic conditions [5,6]. Furthermore, phenol could also be degraded under fermenting conditions, resulting in methane production [7].

One opportunity to overcome this limitation is the provision of electrodes as inexhaustible sources of electron donors and acceptors within the scope of microbial electrochemical technology (MET), an emerging platform for pollutant removal [8,9]. The corresponding reactors are termed bioelectrochemical systems (BES) that harness electroactive microorganisms (EAM), being capable of extracellular electron transfer (EET) and coupling their metabolism with the electric current flow at electrodes [10]. Multiple studies have reported phenol degradation in BESs under anaerobic [[11], [12], [13]] and micro-aerobic conditions [14]. Previous studies on microbial electrochemical phenol degradation analyzed different inoculum sources [15], phenol-degrading microbial communities [16], various electrode materials [17,18], and the influence of the applied anode potential [14].

**Fig. 1:**
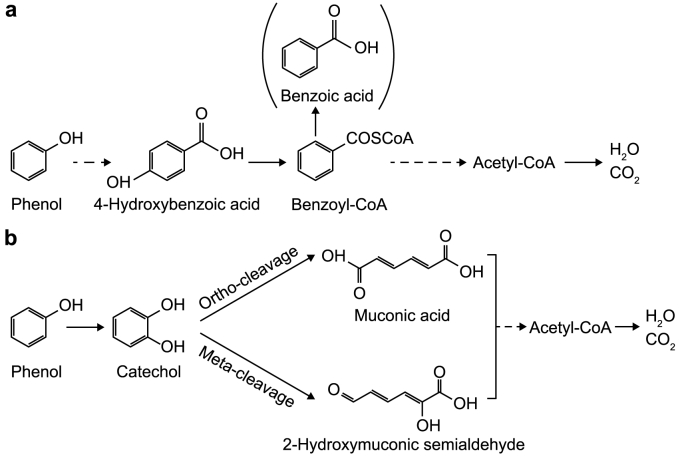
Simplified anaerobic (**a**) and aerobic (**b**) phenol degradation pathways. During anaerobic phenol degradation, phenol is first converted by phenylphosphate synthase and phenylphosphate carboxylase to 4-hydroxybenzoic acid, which is subsequently activated to benzoyl-CoA [19]. The aromatic ring is opened by a hydrolase in the benzoyl-CoA degradation pathway resulting in 3-hydroxypimelyl-CoA being further transformed to acetyl-CoA [22]. Benzoic acid is an artificial decay product of benzoyl-CoA which is commonly detected in corresponding measurements. The dashed line arrows represent multi-step reactions. During aerobic phenol degradation, phenol is first activated by monooxygenases resulting in catechol, followed by cleavage of the benzene ring by dioxygenases either between the two hydroxyl groups (ortho-cleavage) or next to one of the hydroxyl groups (meta-cleavage) [26]. The products are further oxidized into acetyl-CoA utilized in the citric acid cycle [27].

Phenol degradation utilizing an anode as the sole terminal electron acceptor (TEA) is anticipated to follow the common anaerobic phenol degradation pathway. Here, phenol is initially carboxylated, forming 4-hydroxybenzoate as a stable intermediate. This intermediate is then further processed through the reduction and opening of the aromatic ring by the benzoyl-CoA reduction pathway [19] (Fig. 1b). However, corresponding literature concerning phenol degradation in BESs is inconsistent. For instance, muconic acid and catechol, which are typical intermediates for aerobic phenol degradation [5] (Fig. 1a), were reported during assumed anaerobic phenol degradation in BESs [20,21]. Hassan and colleagues reported that chlorophenol and phenol were transformed into benzene in an anaerobic BES [22], which has never been observed before and is incompatible with the known anaerobic phenol degradation pathway [19]. Two studies presented genetic evidence for an anaerobic phenol degradation pathway, but one study suggested a combined anaerobic and aerobic phenol degradation as genes for both pathways were identified [23]. Espinoza-Tofalos and colleagues reported a decreased coverage of the marker gene for the anaerobic phenol degradation (encoding for 4-hydroxybenzoate decarboxylase) for the cultivated microbiome compared to the inoculum [24], questioning the main pathway being responsible for phenol degradation. In another study, 4-hydroxybenzoic acid, a key intermediate for the anaerobic phenol oxidation, was identified, but significant amounts of nitrate, sulfate, Fe(III), and oxygen — all representing alternative TEA — were present in the experiments [25]. Therefore, it is still doubtful and lacks conclusive evidence if the anaerobic phenol degradation in BESs by mixed cultures and using anodes as sole TEAs is feasible and follows the well-known anaerobic phenol degradation pathway.

This study aims to address this knowledge gap by investigating the feasibility of a strict anaerobic phenol degradation process using anodes as sole TEAs. In addition, we evaluated the influences of reactor configuration (one-chamber and two-chamber BESs) and applied anode potential on phenol degradation. Applying high-performance liquid chromatography (HPLC), gas chromatography-mass spectrometry (GC-MS), and amplicon sequencing showed strong evidence that phenol was solely anaerobically degraded. To our knowledge, this is the first report about anaerobic phenol degradation in BESs using anodes as sole TEAs.

## 2 Material and methods

### 2.1 General remarks

All chemicals used in the study were of analytical quality and purchased from Sigma-Aldrich (USA), Merck (Germany), and Carl Roth (Germany). All provided potentials refer to the standard hydrogen electrode (SHE) by conversion from Ag/AgCl sat. KCl reference electrodes (+0.197 V vs. SHE).

### 2.2 Reactor setup, inoculation, and operation

Experiments were performed in BESs consisting of four-neck round-bottom flasks in one-chamber or two-chamber configurations by inserting a tailor-made glass tube to establish an ionic contact via a cation exchange membrane (Fig. S1). Both anode and cathode were graphite electrodes (geometrical surface area 13.4 cm^2^) and a Ag/AgCl sat. KCl reference electrode was used (see SI-1 for experimental details). All reactors were filled with 250 mL modified mineral salt medium (MSM, SI-2). Subsequently, 4 mL of a phenol-degrading enrichment culture (see SI-3 for details) and 0.5 mL of an anaerobic phenol stock solution (47 g L^−1^, leading to an initial concentration, $c_{phenol,0}=$ 90.6 ± 1.9 mg L^−1^) was anaerobically added in an anaerobic chamber (95% N_2_, 5% H_2_ gas atmosphere; Gloveless Anaerobic Glove Box, COY Laboratory Products Inc., USA). Notably, the applied phenol concentration likely did not limit the used phenol-degrading enrichment culture as it is well below reported inhibiting phenol concentrations [27,28]. All reactors were operated at 30 °C and stirred at 400 rpm using a magnetic stirrer. Chronoamperometry (sampling interval 600 s) was performed using a multipotentiostat (MPG-2, Bio-Logic Science Instruments, France). The experimental design comprised one-chamber reactors poised to a working electrode potential of 0.4 V (OC4) and two-chamber reactors poised to 0.4 V (TC4) and 0.2 V (TC2). All experiments were performed in independent triplicates. An additional one-chamber reactor (anode potential 0.4 V) was repeatedly fed with phenol for the accumulation and identification of intermediates of the phenol degradation pathway. Control experiments were identical to the performed bioelectrochemical experiments but without applied potential (open circuit voltage control) or without the addition of the inoculum (abiotic control).

### 2.3 Sampling and sample analyses

Reactors were anaerobically sampled in an anaerobic chamber every 2–3 days. Phenol concentration was measured by HPLC (Section 2.4), the pH was monitored using a pH meter (SevenCompact S220, Mettler Toledo GmbH, Germany), and intermediates were identified using HPLC and GC-MS (Section 2.4).

Biofilm and planktonic biomass samples were taken at the end of each experiment for microbial diversity analysis via amplicon sequencing of 16S rRNA genes (SI-4).

### 2.4 Calculations and statistic

The coulombic efficiency (CE) is the ratio of transferred charge to the anode and the charge corresponding to the consumed substrate, assuming that phenol is fully mineralized to CO_2_ and H_2_O (equation (1)):

(1) $$CE=\frac{\int_{0}^{t}Idt}{F\times \Delta n_{phenol}\times z_{phenol}}$$

where I is current, t is time, F is the Faraday constant (96,485.3C mol^−1^), ${\Delta n}_{phenol}$ is the change in the amount of phenol; $z_{phenol}$ is the number of released electrons assuming phenol is completely mineralized ($z_{phenol}$ = 28).

The phenol degradation rate ($r_{phenol}$, mg L^−1^ d^−1^) was calculated according to equation (2):

(2) $$r_{phenol}=\frac{{\Delta c}_{phenol}}{\Delta t}$$

where ${\Delta c}_{phenol}$ is the change in phenol concentration, and Δ*t* is the time interval.

Experimental data are presented as means with a 95% confidence interval (CI) of the mean. Independent two-sample *t*-tests (similar variances, two-tailed distribution) were performed with a significance level of *α* = 0.95. Normal distribution was verified by applying the Kolomogorov-Smirnov test.

### 2.5 Chemical analysis

Phenol degradation was monitored by HPLC analysis using a ZORBAX Eclipse XDB-C8 column (4.6 × 150 mm, 5-μm) on an Agilent 1100 Series G1316A device. Samples were taken directly from BESs, centrifuged (10,000 g), filtered (0.2 μm PTFE, Macherey-Nagel GmbH & Co.KG, Germany), and subjected to HPLC analysis without further dilution. The detector was set at 208 nm, and peak areas were integrated automatically. Measurements were carried out at room temperature for 30 min. The column was re-equilibrated for 5 min between each sample to the starting measurement conditions. Constituents of the mixture were identified using retention times of pure reference compounds and quantified with external standards. Thus, an 8-point calibration in the range of 0.1–50.0 mg L^−1^ was performed for each standard compound. The mobile phase was prepared daily and degassed for 5 min in an ultrasonic bath before use. HPLC analysis was performed with the gradient shown in Table 1, with a constant flow of 0.8 mL min^−1^. The injection volume was 20 μL.

**Table 1:** HPLC gradient flow with pure acetonitrile +0.01% H_2_SO_4_ (solvent A) and double distilled water + 0.01% H_2_SO_4_ (solvent B).

Time (min)	Solvent A (%)	Solvent B (%)
0	2.0	98.0
3	2.0	98.0
25	95.0	5.0
29	95.0	5.0
30	2.0	98.0

To identify phenol degradation intermediates, derivatized (see below) samples were analyzed via GC-MS. Samples were taken directly from the continuous phenol-fed BESs after the third phenol addition (0.5 mL of the phenol stock solution), centrifuged (10,000 g, room temperature, Sigma 2-16 KL, Sigma Laborzentrifugen GmbH, Germany), filtered (0.2 μm PTFE), and fully dried in a vacuum concentrator for 12 h.

80 μL methoxyamine hydrochloride in pyridine (20 mg mL^−1^, freshly prepared) was added to a dried sample and shaken for 1.5 h at 30 °C, 750 rpm (ThermoMixer C, Eppendorf SE, Germany). The sample was centrifuged for 5 min. Afterward, 80 μL MSTFA (N-Methyl-N-(trimethylsilyl)trifluoroacetamide) was added, and the sample was shaken for 30 min at 37 °C and 750 rpm. Subsequently, the sample was centrifuged as described above for 5 min, and the supernatant was transferred to a GC vial with a micro insert. In addition, a vial with MSTFA was prepared to pre-condition the GC-MS system.

For analysis, 1 μL sample was injected into a GC-MS (GC 7890A and MSD 5975C InertXL, Agilent, Santa Clara, USA) equipped with an HP-5MSI capillary column (30 m × 250 μm × 0.25 μm, Agilent, Santa Clara, USA); helium (1 mL min^−1^) was used as carrier gas. The injector was operated in a split mode (1:10) at a temperature of 250 °C. The initial temperature was 40 °C (held for 1 min) and continuously increased to 70 °C (held for 1 min) by a temperature ramp of 15 °C min^−1^. Afterward, the temperature was increased to 320 °C (ramp of 6 °C min^−1^) and held for 10 min at 320 °C. The compounds in the mixture were identified using the NIST17 mass spectral library.

## 3 Results and discussion

### 3.1 Influence of reactor configuration and applied anode potential on phenol degradation

**Fig. 2:**
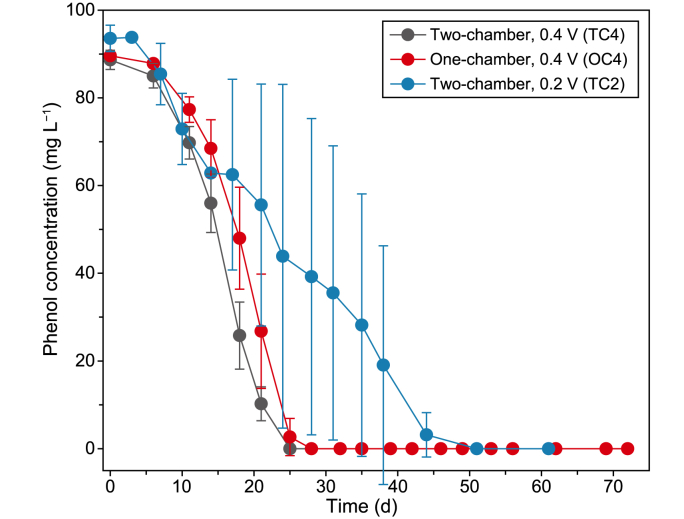
Time course of phenol concentrations ($c_{phenol}$) during chronoamperometric cultivation. The black, red, and blue lines represent TC4 (two-chamber reactors, anode potential 0.4 V), OC4 (one-chamber reactors, 0.4 V), and TC2 (two-chamber reactors, 0.2 V), respectively. The data are mean values, and error bars represent 95% confidence interval (*n* = 3).

Phenol ($c_{phenol,0}$ = 90.6 ± 1.9 mg L^−1^) was degraded entirely in all bioelectrochemical systems (BESs) within 44 days (Fig. 2). Only 2.7 ± 0.8% of phenol was removed during the first seven days, indicating an adaption phase after inoculation (Fig. 2). The phenol removal rate ($r_{phenol}$) increased after seven days in all experiments with an average $r_{phenol}$ of 3.6 ± 0.1, 3.5 ± 0.2, and 2.6 ± 0.9 mg L^−1^ d^−1^ in TC4, OC4, and TC2, respectively, without significant differences (Table 2). Phenol was fully removed within 28 days in TC4 and OC4. In TC2, complete depletion was achieved after 44 days, presumably due to the lower applied anode potential. Moreover, the replicates of TC2 showed a heterogeneous behavior indicated by the high 95% confidence intervals, which were not observed for TC4 and OC4 (Fig. 2). Abiotic and open circuit voltage control reactors showed no phenol removal demonstrating a clear relation between anaerobic phenol degradation and current production (SI-5). The observed $r_{phenol}$ was considerably lower than previously reported values [[29], [30], [31]]. However, the present study did not aim at high performance but to study the feasibility and mechanisms of anaerobic phenol degradation using anodes as sole TEAs. For instance, an $r_{phenol}$ of 12.2–37.0 mg L^−1^ d^−1^ were reported for a BES poised to 0.2 V using an anode area-to-reactor volume ratio (determining the availability of the electron acceptor) of 0.4 cm^2^ mL^−1^ [31], which is eight times higher than in this study (0.05 cm^2^ mL^−1^).

**Table 2:** Overview of performance parameters of BESs.

Reactor configuration	Anode potential vs. SHE (V)	Abbreviation	Average current density (mA cm^−2^)	Coulombic efficiency^a^ (%)	Phenol degradation rate^b^ (mg L^−1^ d^−1^)	pH
Two-chamber	0.4	TC4	0.005 ± 0.001	52.9 ± 9.1	3.55 ± 0.08	6.8 ± 0.2
One-chamber	0.4	OC4	0.019 ± 0.005	180.7 ± 38.8	3.46 ± 0.20	7.3 ± 0.1
Two-chamber	0.2	TC2	0.006 ± 0.003	66.4 ± 27.8	2.59 ± 0.93	6.8 ± 0.2

a Independent two-sample *t*-tests indicated significant differences between TC4/OC4 (*p* = 0.01) and OC4/TC2 (*p* = 0.02) but not between TC4 and TC2 (*p* = 0.50).

b Independent two-sample *t*-tests indicated no significant differences between TC4/OC4 (*p* = 0.72), OC4/TC2 (*p* = 0.14), and TC4/TC2 (*p* = 0.06).

Although TC4 and OC4 exhibited no significant differences in $r_{phenol}$, their microbial composition differed noticeably (Section 3.3). The achieved average current density and CE were considerably higher in OC4 (Table 2, Fig. S3) than in TC4 and TC2, which can be assigned to the microbial utilization of cathodically produced H_2_ and thus recurring recycling of electrons as it was demonstrated before [32,33]. Intriguingly, the CE of two-chamber BES is high (Table 2) when compared to the literature (CE of 4–23% [21]). Considering the share of electrons required for biomass formation and maintenance, the high CE illustrates that phenol was most likely fully mineralized and indicates a negligible role of other terminal electron acceptors (including oxygen) than the anode.

### 3.2 Identification of phenol degradation intermediates in bioelectrochemical systems

**Fig. 3:**
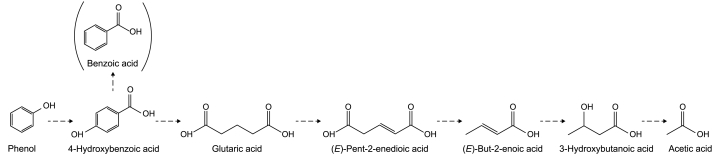
The proposed route of the anaerobic phenol degradation in BESs using anodes as sole terminal electron acceptor based on compounds annotation by HPLC and GC-MS analysis [22]. Benzoic acid is an artificial decay product of benzoyl-CoA, which is commonly detected in corresponding measurements, as in the here performed HPLC analysis. The dashed arrows indicate potential one- and multi-step reactions.

During reactor operation, minor peaks of benzoic acid and 4-hydroxybenzoic acid were observed by HPLC in all BESs (Fig. S4), suggesting no differences in the phenol degradation pathway between one-chamber and two-chamber reactors as well as between the different applied anode potentials. This also indicated that phenol was anaerobically degraded in the BES, as these compounds are typical metabolites of the anaerobic phenol degradation pathway [34]. To verify the identity and occurrence of these two intermediates, an additional one-chamber BES was inoculated using the same inoculum. After being repeatedly fed with phenol, this long-term BES showed an accumulation of the intermediates mentioned above (Fig. S5). Furthermore, GC-MS analysis (Fig. S6) revealed the presence of glutaric acid, (*E*)-pent-2-enedioic acid, (*E*)-but-2-enoic acid, and 3-hydroxybutanoic acid, which are typical downstream metabolites of the anaerobic phenol degradation pathway [19,26,35,36]. Fig. 3 highlights the metabolites annotated using HPLC and GC-MS measurements of the long-term BES. 10.13039/100014337Furthermore, the absence of metabolites being typical for the aerobic phenol degradation pathway strongly supports the hypothesis that microorganisms solely degraded phenol via the anaerobic degradation pathway using anodes as sole TEAs. Although the literature about characteristics of phenol degradation at controlled microaerobic conditions is unavailable to our knowledge, for degradation of other monoaromatics at microaerobic conditions, it has been observed that metabolites of the initial oxygen-dependent mono- or dioxygenation reactions at the aromatic ring are usually accumulating in the external medium, e.g., (chloro)catechols [[37], [38], [39]].

### 3.3 Microbial community composition

At the end of the experiments, anode biomass and planktonic cells were analyzed via 16S rRNA gene amplicon sequencing to reveal structure-function relationships within the bacterial community. Analyzing the genetic data with principal coordinates analysis (PCoA) indicated that each experimental condition led to different microbial communities. One-chamber and two-chamber reactors differed more than two-chamber reactors poised at different anode potentials (Fig. S7). Whereas different anode potentials influence the EET rate and, presumably, the abundance of weak and strong electricigens (see discussion below), the reactor configuration determines the availability of cathodically produced H_2_. The presence of this additional substrate certainly influenced the microbial composition, as it was already demonstrated for acetate-fed electroactive biofilm anodes [40,41].

#### 3.3.1 Microorganisms directly involved in anaerobic phenol degradation using anodes as sole terminal electron acceptors

**Fig. 4:**
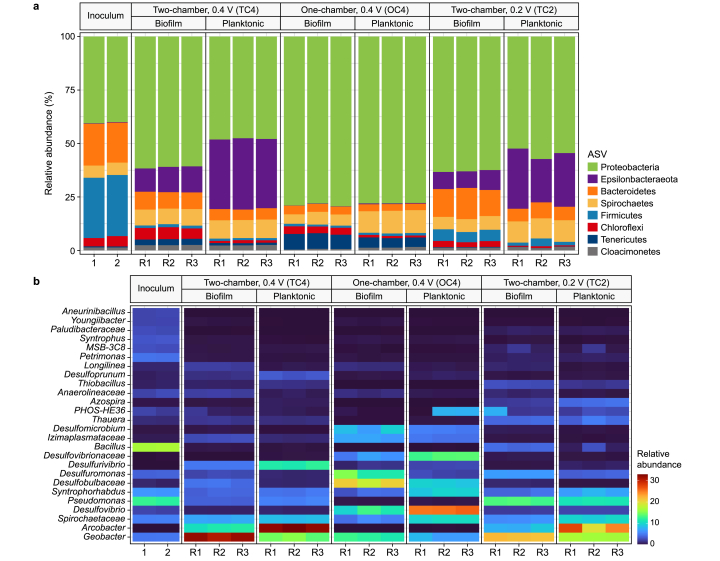
Community composition of microbial samples derived from the inoculum (SI-3), biofilm, and planktonic cells at the phylum (**a**) and genus (**b**) level. R1, R2, and R3 are independent replicates of each experimental condition.

Proteobacteria dominated all BESs with a relative abundance of 63.1 ± 11.2% (combined value of anode biomass and planktonic biomass), representing a considerable enrichment compared to the inoculum (39.3%) (Fig. 4a). The relative abundance of Epsilonbacteraeota significantly increased in both two-chamber reactors, especially in the planktonic phase (28.4 ± 4.7%), while they were nearly absent in one-chamber reactors. Notably, Firmicutes (i.e., *Bacillus*) were less abundant in BESs than in the inoculum (Fig. 4a and b). Overall, the results confirm previous studies, showing that Proteobacteria are the dominant phylum in BES treating phenol [13,42] and phenolic compounds [23].

On the genus level, the relative abundances of phylotypes belonging to *Geobacter*, being one model genus for direct EET, were significantly higher (see SI-6 for an overview of statistical tests on microbial community composition) in two-chamber reactors (biofilm: 26.6 ± 4.9%, planktonic cells: 15.7 ± 1.1%) than one-chamber reactors (biofilm: 12.1 ± 0.6%, planktonic cells: 7.1 ± 0.7%) (Fig. 4b). In one-chamber reactors, the presence of hydrogen as an additional electron donor, alongside acetate, led to a higher microbial diversity, as hydrogen utilization is a widespread ability of anaerobes [43,44]. *Geobacter* species can directly interact with electrodes leading to current production [45]. The involvement of two different *Geobacter* phylotypes in the anaerobic phenol degradation is hypothesized to occur through two distinct mechanisms. First, complete phenol oxidation is performed by *Geobacter metallireducens,* which anaerobically degrades phenol by performing EET [34]. Second, *Geobacter* phylotypes performed EET based on oxidizing acetate and other small carbon molecules that originated from fermentative anaerobic phenol degradation performed by, for instance, *Syntrophorhabdus*. This genus hosts syntrophic phenol degraders producing, e.g., acetate and butyrate [46,47], and its occurrence in phenol-degrading BESs was already reported [48]. However, its relative abundance was comparably low (5.6 ± 2.1% in all experiments), with a slightly higher abundance in planktonic biomass (7.1 ± 2.0%). *Syntrophorhabdus aromaticivorans* were reported to exhibit slow growth under syntrophic conditions with phenol as the substrate, likely due to the ATP-consuming activation of phenol by phenylphosphate synthase [7]. Its appearance in the inoculum (6.3%), supplemented with phenol but with different TEA (i.e., sulfate), suggests the particular role of phylotypes affiliated to *Syntrophorhabdus* in anaerobic phenol degradation, providing metabolites to further members of the microbial metabolic network (i.e., mutualistic metabolism). Furthermore, a phylotype related to *Arcobacter* (i.e., Epsilonbacteraeota), a genus known for containing members capable of manganese oxide reduction coupled to acetate oxidation [49] and previously identified at the anode of BESs [50], could contribute to the degradation of intermediates and current production. It was enriched in the two-chamber BES (biofilm: 9.7 ± 1.7%, planktonic cells: 28.2 ± 4.8%) but not in the one-chamber BES. Furthermore, phylotypes affiliated with the genus *Pseudomonas* could contribute to phenol degradation. They were present in two-chamber reactors (8.3 ± 4.0%) but exhibited a negligible relative abundance in one-chamber reactors (0.3 ± 0.0%). *Pseudomonas-*affiliated phylotypes were described as phenol degraders in the anaerobic BES [15,51] and for aerobic conditions [52]. The closely related genus *Thauera* was also reported for anaerobic phenol degradation [19] and was slightly increased in TC2 (2.8 ± 1.4%). Notably, *Pseudomonas* and *Thauera*-affiliated phylotypes from activated sludge were shown to assimilate phenol under anaerobic conditions [53]. However, it remains unclear if *Pseudomonas* oxidized phenol or only consumed secreted small carbon molecules derived from phenol oxidation by other microbial community members and then performed mediated EET [54]. *Pseudomonas* is also considered a weak electricigen, exhibiting low currents typically during redox stress and having limited growth while performing EET [55]. In our study, the weak electricigenity of *Pseudomonas* is indicated by the observation that its relative abundance was significantly higher (e.g., biofilm: 13.3 ± 0.3% in TC2 and 3.6 ± 0.1% in TC4, *p* = 1 × 10^−6^) at a lower anode potential (i.e., less driving force for EET). At the same time, the relative abundance of *Geobacter*, a strong electricigen, was significantly lower in TC2 compared to TC4 (e.g., biofilm: 31.4 ± 0.7% in TC4 and 21.7 ± 0.4% in TC2, *p* = 4 × 10^−5^). At lower anode potentials, weak electricigens could occupy an ecological niche alongside the strong electricigens from the genus *Geobacter*. But at higher anode potentials, *Geobacter* outcompeted weak electricigens due to their superior EET capabilities, high affinity, and high uptake rate for acetate, one central intermediate of the anaerobic phenol degradation [26,33,56].

#### 3.3.2 Microorganisms involved in non-bioelectrochemical processes

One-chamber reactors exhibited a higher relative abundance of genera involved in sulfur cycling, including *Desulfovibrio*, uncultured *Desulfobulbaceae*, *Desulfuromonas*, *Desulfurivibrio*, *Desulfoprunum*, and *Desulfomicrobium* (52.7 ± 1.2% averaging biofilm and planktonic cells) compared to two-chamber reactors (12.4 ± 4.1%, *p* = 1 × 10^−13^). For instance, the sulfate-reducing family *Desulfobulbaceae*, comprising physiologically versatile members who, e.g., can degrade aromatics like toluene [57], was detected in a high relative abundance in one-chamber reactors (biofilm: 21.5 ± 1.1%, planktonic cells: 12.4 ± 0.3%). Their presence indicates a contribution to phenol degradation and other processes not directly linked to phenol oxidation. Most of these genera contain members described to utilize H_2_, produced at the cathode, as the electron donor for sulfate reduction [58], which was present in small amounts (1.4 mM) in the medium. Considering this low sulfate concentration, it seems conceivable that the produced sulfide was abiotically oxidized at the anode, as demonstrated previously [59]. Consequently, sulfide oxidation and sulfate recycling could contribute to the high *CE* in one-chamber reactors and a continual enrichment of genera involved in sulfur cycling, respectively. An additional trophic layer could be constituted by *Spirochaetaceae,* which exhibited a relative abundance of 7.6 ± 2.6% and 7.8 ± 1.4% in one-chamber and two-chamber reactors, respectively. *Spirochaetaceae*, frequently identified in anaerobic hydrocarbon-contaminated environments, were supposed to recycle necromass secreting small electron donors and other nutrients to the microbial metabolic network [60]. The absence of any gas overpressure in all reactors, the emergence of *Geobacter* and *Arcobacter* (in two-chamber reactors), and genera involved in sulfur cycling (in one-chamber reactors) indicate a minor role of methanogens. Presumably, they are outcompeted by the genera mentioned above, as using an electrode and sulfate as the TEA is energetically more favorable than CO_2_.

## 4 Conclusion

In contrast to inconclusive literature, this work provides evidence for strict anaerobic phenol degradation using anodes as sole terminal electron acceptors. Our results demonstrated the presence of typical intermediates of anaerobic degradation, such as 4-hydroxybenzoic acid and pimelic acid, whereas specific metabolites for aerobic phenol degradation, including catechol and muconic acid, were absent. The removal rate was comparable in all tested reactor configurations, with the highest observed in one-chamber reactors at 0.4 V, achieving a phenol removal rate of 3.6 ± 0.1 mg L^−1^ d^−1^. *Syntrophorhabdus*, a genus containing a syntrophic phenol degrader, was identified in all experiments, and the inoculum with a stable relative abundance of 7.1 ± 2.0%, suggesting a key role for anaerobic phenol degradation, also in bioelectrochemical systems. Subsequently, the consumption of intermediates, such as acetate by *Geobacter* (relative abundance of 26.6 ± 4.9% and 15.7 ± 1.1% in biofilm and planktonic phase, respectively) and *Arcobacter* (relative abundance of 9.7 ± 1.7% and 28.2 ± 4.8% in biofilm and planktonic phase, respectively) in two-chamber reactors, resulted in current production. In one-chamber reactors, the lower relative abundance of known electroactive microorganisms is accompanied by a strong emergence of genera involved in sulfur cycling, with a relative abundance of 52.7 ± 1.2%, suggesting their contribution to phenol oxidation or parallel metabolic processes. However, further investigations employing more detailed experiments with pure cultures, defined co-cultures, and multi-omics approaches are necessary to reveal a potential mutualistic metabolism and microbial interactions associated with anaerobic phenol degradation.

## CRediT author contribution statement

**Shixiang Dai:** Methodology, Investigation, Data Curation, Writing - Original Draft. **Falk Harnisch:** Conceptualization, Supervision, Writing - Review & Editing. **Micjel Chávez Morejón:** Investigation, Data Curation, Writing - Review & Editing. **Nina Sophie Keller:** Investigation, Data Curation, Data Analysis, Writing - Review & Editing. **Benjamin Korth:** Conceptualization, Supervision, Methodology, Writing - Review & Editing. **Carsten Vogt:** Conceptualization, Supervision, Writing - Review & Editing.

## Data availability statement

The 16S rRNA amplicon sequence reads of this study were deposited in the European Nucleotide Archive (ENA) database under accession No. PRJEB56377.

## Declaration of competing interest

The authors declare that they have no known competing financial interests or personal relationships that could have appeared to influence the work reported in this paper.
