# Supplementary material for: Microbial electricity-driven anaerobic phenol degradation in bioelectrochemical systems
Source: Environ Sci Ecotechnol. 2023 Jul 26;17:100307. doi: 10.1016/j.ese.2023.100307 (PMC10432169; doi:10.1016/j.ese.2023.100307)
Supplement: Multimedia component 1 [file mmc1.pdf]

Supplementary Information

Microbial electricity-driven anaerobic phenol degradation in bioelectrochemical systems

Shixiang Dai[1], Falk Harnisch[1], Micjel Chávez Morejón[1], Nina Sophie Keller[2], Benjamin Korth[1]*, Carsten Vogt[2]*

[1] Department of Environmental Microbiology, Helmholtz Centre for Environmental Research GmbH - UFZ, Leipzig, Germany

[2] Department of Isotope Biogeochemistry, Helmholtz Centre for Environmental Research GmbH - UFZ, Leipzig, Germany

*Corresponding author: benjamin.korth@ufz.de, carsten.vogt@ufz.de

SI-1. Experimental details

All microbial electrochemical experiments were carried out in bioelectrochemical systems (BES) consisting of four-neck round-bottom flasks (Lenz Laborglas GmbH & CO.KG, Germany) filled with 250 mL of a modified mineral salt medium (MSM, see Table S1) representing one-chamber configuration. The two-chamber configuration was created by inserting a 50 mL tailor-made glass tube as cathode chamber being ionically connected to the anode chamber via a cation exchange membrane (CEM, fumasep®FKE, FuMA-Tech GmbH, Germany) (Figure S1), which was fixed with an O-ring and an aluminum cap (Figure S1). The cathode chamber was filled with 40 mL of modified MSM (SI-2). Both anode (working electrode, WE) and cathode (counter electrode, CE) consisted of a graphite rod (diameter: 1 cm, length: 4 cm, area: 13.35 cm^2, quality CP-2200, CP-Graphitprodukte GmbH, Germany) electronically connected to a stainless steel wire (Goodfellow GmbH, Germany) which was covered by a polytetrafluoroethylene tube (Shrink-Kon®, Thomas & Betts Corp., U.S.A.). An Ag/AgCl reference electrode (SE11, Xylem Analytics Germany Sales GmbH & Co. KG Sensortechnik Meinsberg, Germany) was pierced through a chloroprene stopper (Deutsch & Neumann GmbH, Germany) and connected to the anode chamber.

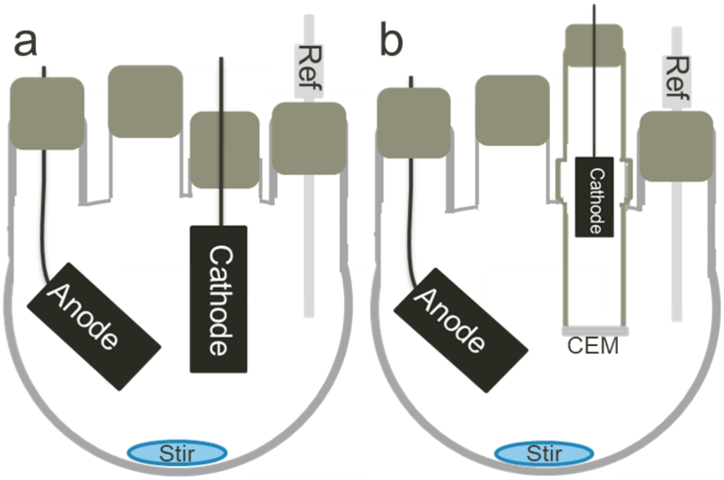

Figure S1. Sketch of bioelectrochemical systems. a) one-chamber reactor with both anode and cathode being immersed in the same electrolyte solution and b) two-chamber reactor with anode and cathode being separated by a cation exchange membrane (CEM). Stir: Stirrer, Ref: Ag/AgCl sat. KCl reference electrode.

SI-2 Composition of the modified mineral salt medium (MSM)

The composition of the modified mineral salt medium (MSM) is listed below (Table S1). Components were dissolved in ca. 960 mL distilled water, autoclaved (121 °C, 20 min) and purged with N_2 afterwards. Stock solutions (trace elements, vitamins, $NaHCO_3$) were sterilized by autoclaving (121 °C, 20 min) or filtration (vitamins). Vitamins and trace elements were purged with N_2 and the $NaHCO_3$ stock solution was purged with CO_2 to ensure anaerobic conditions. Subsequently, all stock solutions were added to the basal medium in an anaerobic chamber. The pH was adjusted to 7 by adding HCl solution (2 mol L^{-1}).

Table S1. Composition of the modified mineral salt medium (MSM).

	Component	Concentration	
Basal medium	NaCl	0.5 g L^{-1}	
	KH_2PO_4	0.5 g L^{-1}	
	NH_4Cl	0.4 g L^{-1}	
	KCl	0.4 g L^{-1}	
	$MgCl_2$	0.5 g L^{-1}	
	Na_2SO_4	0.2 g L^{-1}	
	$CaCl_2$	0.1 g L^{-1}	
The anaerobic supplementary stock solutions were added to the basal medium in an anaerobic chamber.			
Supplementary stock solutions	Volume added	Components	
$NaHCO_3$	30 mL	CO_2-saturated 1 mol L^{-1} $NaHCO_3$ solution	
Vitamin solution	5 mL	4-Aminobenzoe acid	8 mg L^{-1}
		D(+)-Biotine	2 mg L^{-1}
		Nicotinic acid	20 mg L^{-1}
		Ca-D(+)-pantothenate	10 mg L^{-1}
		Pyridoxamine hydrochloride	30 mg L^{-1}
		Thiamine dichloride	20 mg L^{-1}
Trace element solution SL-10	1 mL	HCl (37%)	8.5 mg L^{-1}
		$FeCl_2 \times 4\ H_2O$	1.5 mg L^{-1}
		H_3BO_3	6 mg L^{-1}
		$CoCl_2 \times 6\ H_2O$	190 mg L^{-}
		$MnCl_2 \times 4\ H_2O$	100 mg L^{-1}
		$ZnCl_2$	70 mg L^{-1}
		$Na_2MoO_4 \times 2\ H_2O$	36 mg L^{-1}
		$NiCl_2 \times 6\ H_2O$	24 mg L^{-1}
		$CuCl_2 \times 2\ H_2O$	2 mg L^{-1}
Vitamin B12 solution	1 mL	Cyanocobalamin	50 mg L^{-1}
Selenite-tungsten solution	1 mL	NaOH	0.5 g L^{-1}
		$Na_2SeO_3 \times 5\ H_2O$	3 mg L^{-1}
		$Na_2WO_4 \times 2\ H_2O$	4 mg L^{-1}

SI-3 Preparation of the inoculum for bioelectrochemical systems

A phenol-degrading enrichment culture was cultivated by using sand sediment from a hydrocarbon-contaminated site in the federal state of Saxony-Anhalt, Germany [1]. The sediment samples were immediately put in an anaerobic jar and stored for several years at 4 °C. An enrichment culture was established and maintained since October 2019 by adding 250 mL sediment in 500 mL anaerobic bicarbonate-buffered mineral salt media (MSM) containing 1 mM phenol as substrate at 25 °C. The enrichment culture was re-supplemented with phenol (1 mM). Once phenol had been consumed, the medium was refreshed by removing 300 mL clear supernatant and adding new anaerobic MSM (300 mL) in an anaerobic chamber.

For inoculation of BES, the enrichment culture was gently shaken for 1 min and 100 mL suspended liquid was extracted followed by centrifugation (4 °C, 10,000 g, Sigma 2-16KL, Sigma Laborzentrifugen GmbH, Germany) and re-suspension in 20 mL MSM. Each BES was inoculated with 4 mL of this suspension.

SI-4 Microbial community analysis

Microbial samples were taken at the end of each experiment. The anode biofilm samples from all BES experiments (Table S1) were obtained with a sterile spatula, while planktonic cells were harvested by centrifuging 24 mL reactor liquid (10 min, 4 °C, 10, 000 g). Both pellet and biofilm, were stored at −30 °C until analysis. Genomic DNA from biofilms and planktonic cells was extracted using the DNeasy PowerSoil Kit (Qiagen, Hilden, Germany) following the manufacturer's instructions. Fluorometric quantification of extracted DNA was performed using an Invitrogen ™ Qubit ™ Fluorometer and Qubit ™ dsDNA HS Assay (Thermo Fisher Scientific Inc., U.S.A.) according to manufacturer's instructions.

For amplicon sequencing, the V3 - V4 hypervariable region of the16S rRNA gene was amplified according to reference [3] using 2x MyTaq™ Mix (Bioline, UK) and the primer-set S-D-Bact-341-b-S-17/S-D-Bact-0785-a-A-21 due to its good coverage of bacterial diversity. Sequencing libraries were prepared as described in the Illumina 16S Metagenomic Sequencing Library Preparation protocol [4], whereby, pooled libraries (diluted to 4 nM) were run on an Illumina MiSeq system (Illumina, USA) using V3 600 cycles chemistry. QIIME 2 (version 2019.1) [5] with cutadapt (version 2.10) [6], fastQC (0.11.9, http://www.bioinformatics.babraham.ac.uk/projects/fastqc), multiQC (1.10.1) [7], DADA2 (1.18.0) [8], and SILVA release 132 [9] were used for primer removal, quality control, denoising, i. e., trimming, quality filtering, de-replicating, chimera removal, and merging of reads, and taxonomic assignment of raw and de-multiplexed sequence reads.

Using vegan and phyloseq [10] packages in RStudio version 2022.02.1+461, rarefaction curves were created to assess the sequencing depth for rarefaction using the function rarefy_even_depth(). Curves plateaued at around 10,000 reads; nevertheless, the threshold was set at the minimum number of reads acquired (16,590) to minimize amplicon sequence variant (ASV) loss. Principal coordinates analysis (PCoA), also known as metric multidimensional scaling (MDS), with Bray-Curtis distance measure was performed using the function ordinate() and plotted using the function plot_ordination() (phyloseq and ggplot2 packages). The Bray-Curtis dissimilarity matrix was chosen as it is not affected by zero values between samples, but it was square-root transformed in order to avoid negative Eigenvalues. For significance testing, a Permutational Multivariate

Analysis of Variance (PERMANOVA [11]) was conducted using the function adonis2() (vegan package).

Barplot and heatmap were created with the function ggplot() and ggarrange() (ggplot2, scales, ggh4x, and ggpubr packages) using color palettes from RColorBrewer or viridis package, the melt() function from the reshape2 package [12] to melt the datasets into single column formats.

SI-5 Control experiments

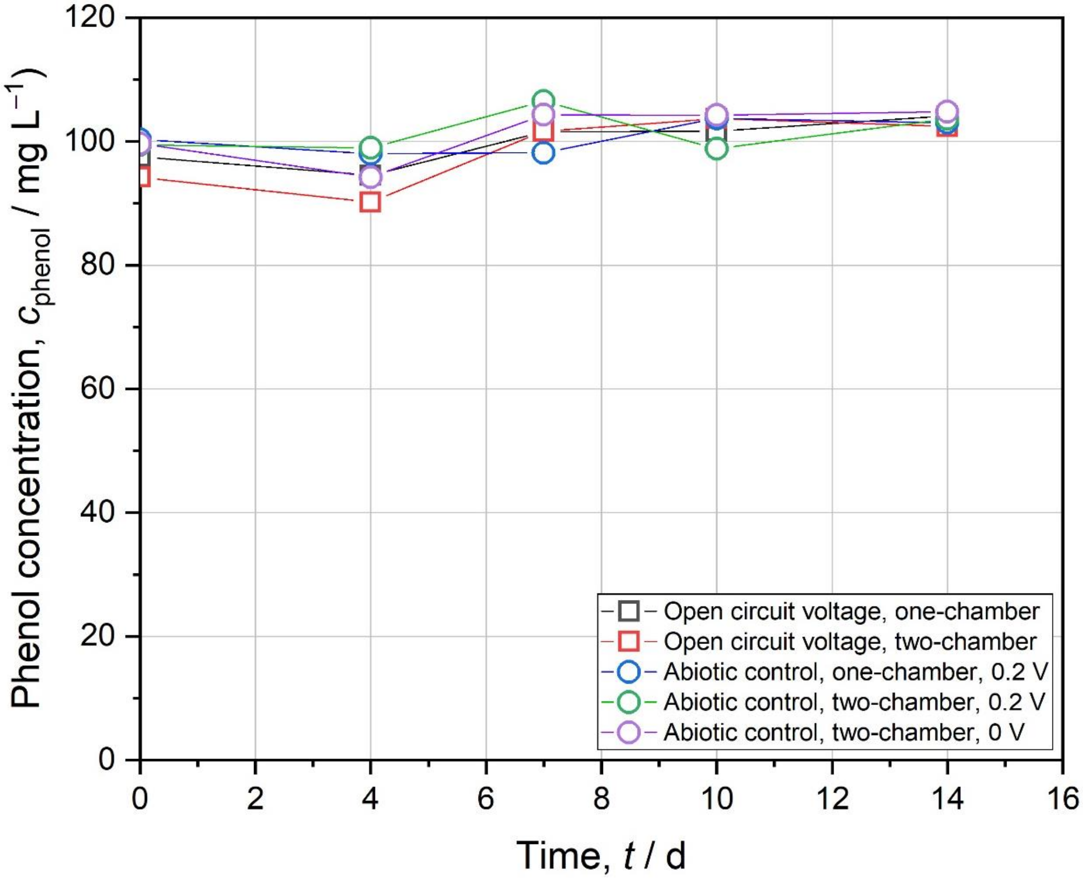

Figure S2. Control experiments showed no phenol degradation. Bioelectrochemical reactors were identical to the performed experiments but without an applied potential (open circuit voltage) or without addition of the inoculum (abiotic control).

SI-6 Statistics

Table S2. Overview about *p*-values of independent two-sample t-tests (similar variances, two-tailed distribution) with the relative amplicon abundances of the genus *Geobacter*.

		Biofilm TC4	Biofilm TC2	Biofilm OC4
Biofilm	TC4		$4\cdot10^{-5}$	$6\cdot10^{-6}$
	TC2			$4\cdot10^{-6}$
	OC4			
		Planktonic cells		
Planktonic cells	TC4		0.02	$4\cdot10^{-4}$
	TC2			$5\cdot10^{-5}$
	OC4			

Table S3. Overview about *p*-values of independent two-sample t-tests (similar variances, two-tailed distribution) with the relative amplicon abundances of the genus *Syntrophorhabdus*.

		Biofilm TC4	Biofilm TC2	Biofilm OC4
Biofilm	TC4		0.61	0.90
	TC2			0.85
	OC4			
		Planktonic cells		
Planktonic cells	TC4		0.003	$2\cdot10^{-4}$
	TC2			0.01
	OC4			

Table S4. Overview about *p*-values of independent two-sample t-tests (similar variances, two-tailed distribution) with the relative amplicon abundances of the genus *Arcobacter*.

		Biofilm TC4	Biofilm TC2	Biofilm OC4
Biofilm	TC4		0.01	$8 \cdot 10^{-6}$
	TC2			$7 \cdot 10^{-5}$
	OC4			
		Planktonic cells		
Planktonic cells	TC4		0.02	$4 \cdot 10^{-8}$
	TC2			$5 \cdot 10^{-4}$
	OC4			

Table S5. Overview about *p*-values of independent two-sample t-tests (similar variances, two-tailed distribution) with the relative amplicon abundances of the genus *Pseudomonas*.

		Biofilm TC4	Biofilm TC2	Biofilm OC4
Biofilm	TC4		$1 \cdot 10^{-6}$	$9 \cdot 10^{-8}$
	TC2			$3 \cdot 10^{-7}$
	OC4			
		Planktonic cells		
Planktonic cells	TC4		$3 \cdot 10^{-6}$	$6 \cdot 10^{-7}$
	TC2			$1 \cdot 10^{-7}$
	OC4			

Table S6. Overview about *p*-values of independent two-sample t-tests (similar variances, two-tailed distribution) with the relative amplicon abundances of genera involved in sulfur cycling (*Desulfovibrio*, uncultured *Desulfobulbaceae*, *Desulfuromonas*, *Desulfurivibrio*, *Desulfoprunum*, and *Desulfomicrobium*).

		Biofilm TC4	Biofilm TC2	Biofilm OC4
Biofilm	TC4		$5{\cdot}10^{-5}$	$1{\cdot}10^{-8}$
	TC2			$3{\cdot}10^{-9}$
	OC4			
		Planktonic cells		
Planktonic cells	TC4		$1{\cdot}10^{-5}$	$2{\cdot}10^{-7}$
	TC2			$6{\cdot}10^{-9}$
	OC4			

Table S7. Overview about *p*-values of independent two-sample t-tests (similar variances, two-tailed distribution) with the relative amplicon abundances of the family *Spirochaetaceae*.

		Biofilm TC4	Biofilm TC2	Biofilm OC4
Biofilm	TC4		0.01	0.01
	TC2			0.11
	OC4			
		Planktonic cells		
Planktonic cells	TC4		$1{\cdot}10^{-3}$	$3{\cdot}10^{-3}$
	TC2			0.26
	OC4			

SI-7 Current production of bioelectrochemical systems

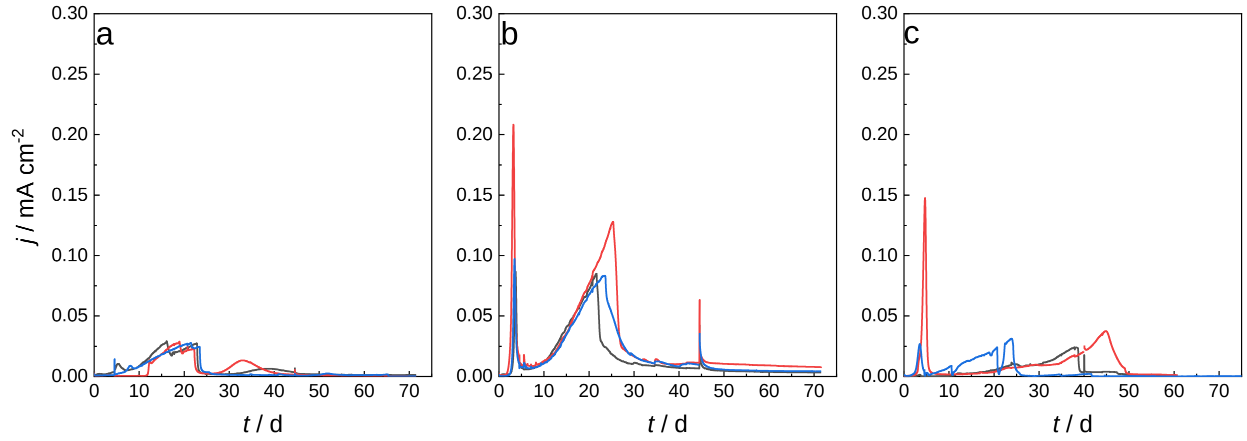

Figure S3. Time course of current densities (*j*) generated in BES. a) TC4: two-chamber reactors, anode potential 0.4 V; b) OC4: one-chamber reactor, 0.4 V; and c) TC2: two-chamber reactor, 0.2 V. Black, blue and red lines represent three replicates of each experimental condition.

SI-8 Results of chemical analyses

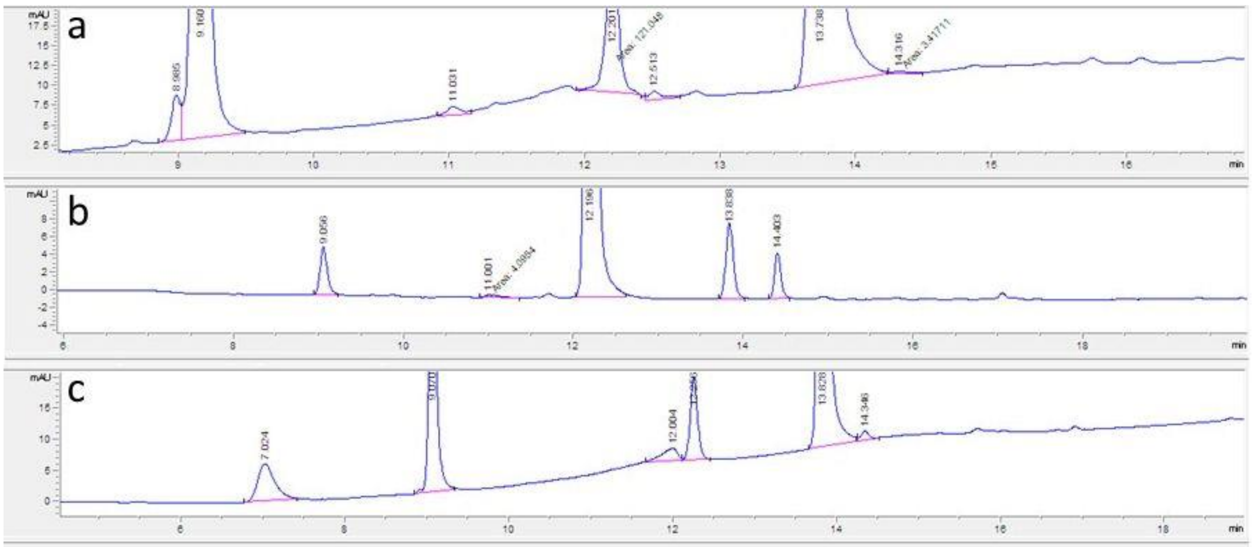

Figure S4. HPLC chromatogram at different detector wavelengths showing minor peaks of benzoic acid and 4-hydroxy-benzoic acid indicating anaerobic phenol degradation in BES. a) OC4: One-chamber reactor, anode potential 0.4 V, detector wavelength 210 nm. 4-hydroxy-benzoic acid and benzoic acid at retention times 11.031 min and 14.316 min, respectively. b) TC2: Two-chamber reactor, anode potential 0.2V, detector wavelength 254.4 nm. 4-hydroxy-benzoic acid and benzoic acid at retention times 11.001 min and 14.403 min, respectively c) TC4: Two-chamber reactor, anode potential 0.4 V, detector wavelength 208.4 nm. Benzoic acid at retention time 14.345 min.

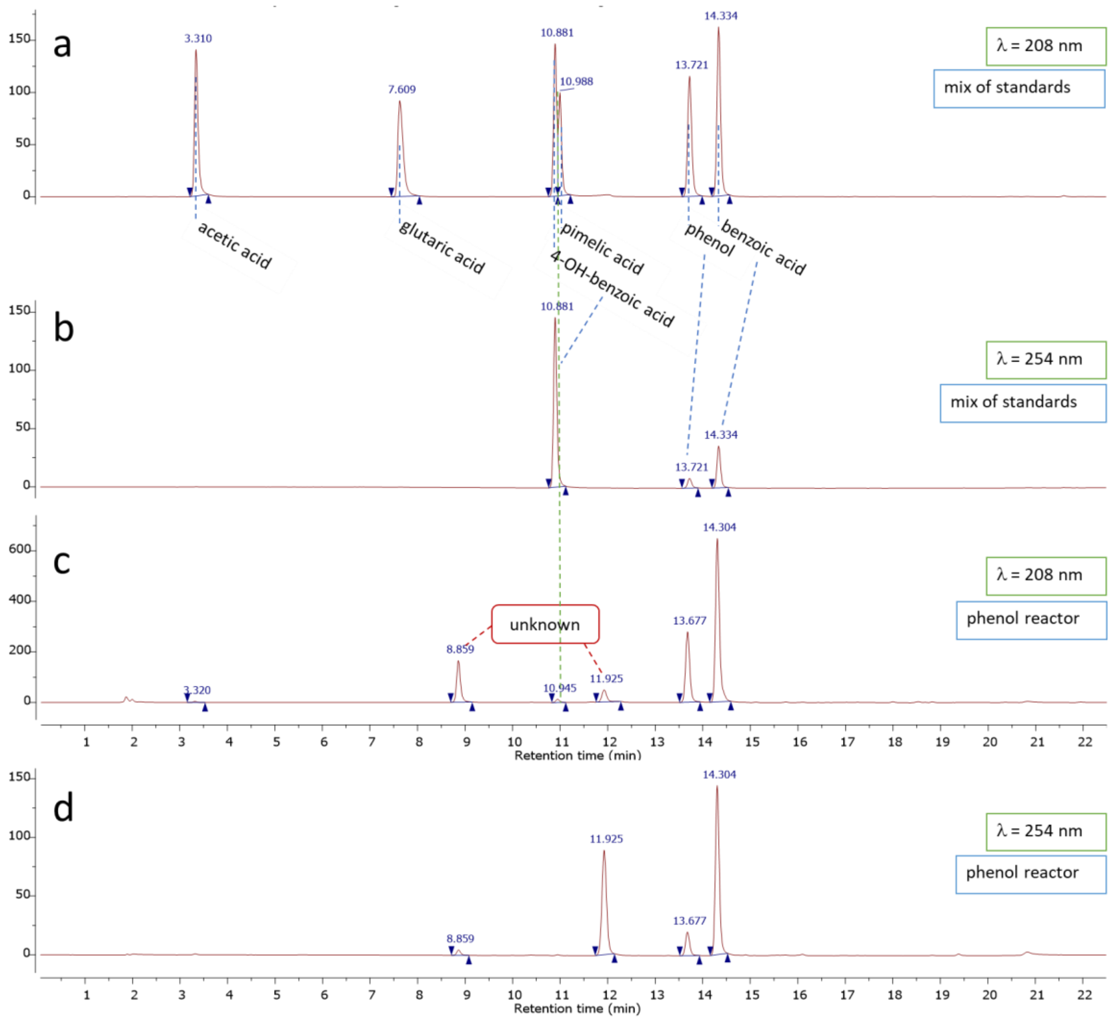

Figure S5. Identification of intermediates of phenol degradation in BES using HPLC. a) HPLC chromatogram at detector wavelength of 208 nm of standard compounds; b) HPLC chromatogram at detector wavelength of 254 nm of standard compounds; c) HPLC chromatogram at detector wavelength of 208 nm of BES sample; d) HPLC chromatogram at detector wavelength of 254 nm of BES sample. The sample measured here was from the continuously phenol-fed BES in one-chamber configuration and sampled after the third phenol addition. Pimelic acid and 4-hydroxy-benzoic acid overlap using the HPLC method at detector wavelength of 208 nm (sub-figure a). In order to differentiate both compounds, another detector wavelength of 254 nm was used, at which only 4-hydroxy-benzoic acid could be detected at 254 nm (sub-figure b). Therefore, those two compounds were separated. A minor peak at the same retention time of pimelic acid and 4-hydroxy-benzoic acid was observed at detector wavelength of 208 nm (sub-figure c), but not at 254 nm (sub-figure d), therefore the peak was assigned to pimelic acid.

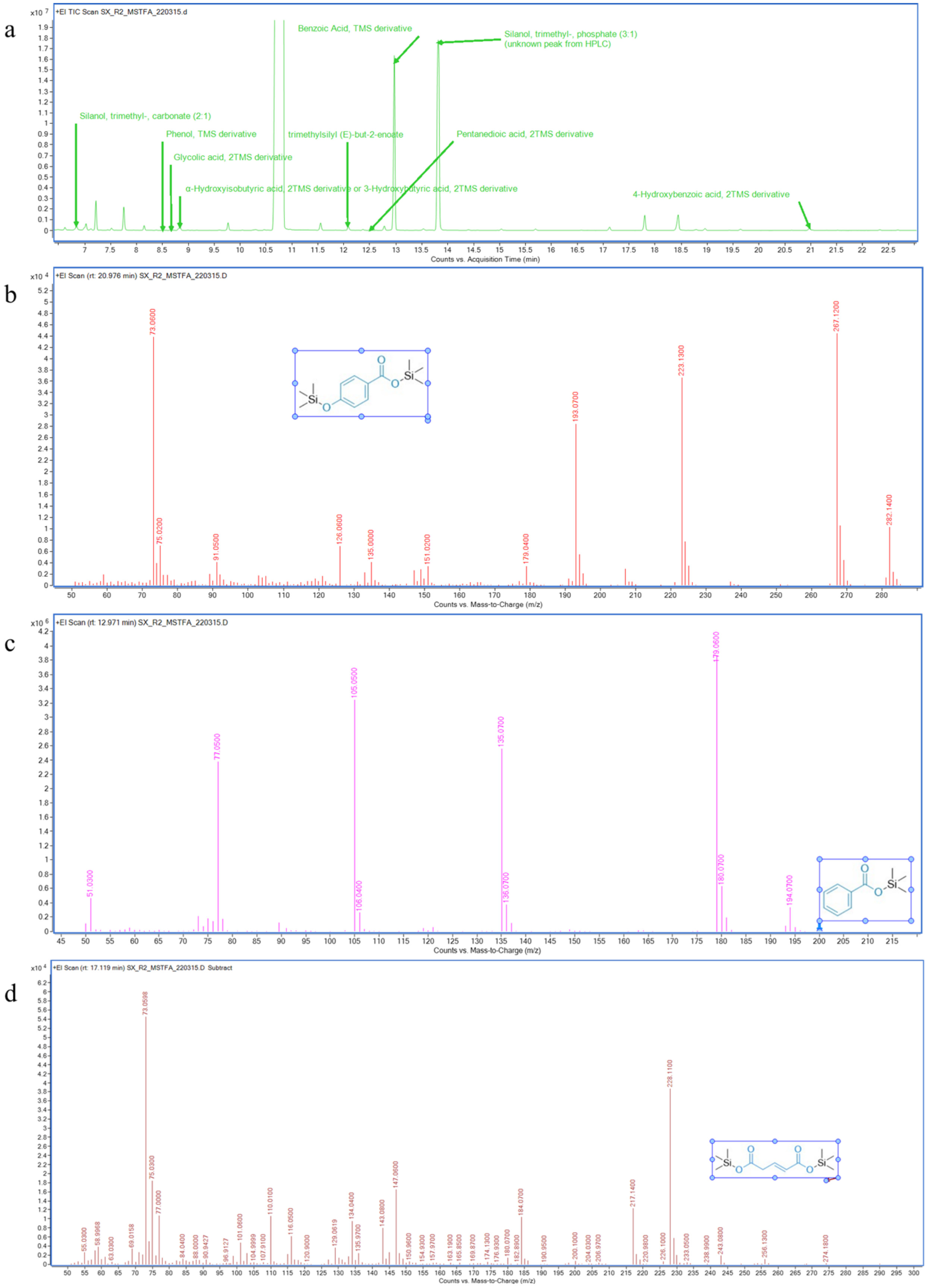
a
+EI TIC Scan SX_R2_MSTFA_220315.d
Silanol, trimethyl-, carbonate (2:1)
Phenol, TMS derivative
Glycolic acid, 2TMS derivative
α-Hydroxyisobutyric acid, 2TMS derivative or 3-Hydroxybutyric acid, 2TMS derivative
Benzoic Acid, TMS derivative
trimethylsilyl (E)-but-2-enoate
Pentanedioic acid, 2TMS derivative
Silanol, trimethyl-, phosphate (3:1)
(unknown peak from HPLC)
4-Hydroxybenzoic acid, 2TMS derivative
Counts vs. Acquisition Time (min)
b
+EI Scan (rt: 20.976 min) SX_R2_MSTFA_220315.D
73.0600
75.0200
91.0500
126.0600
135.0000
151.0200
179.0400
193.0700
223.1300
267.1200
282.1400
Counts vs. Mass-to-Charge (m/z)
c
+EI Scan (rt: 12.971 min) SX_R2_MSTFA_220315.D
51.0300
77.0500
105.0500
106.0400
135.0700
136.0700
179.0600
180.0700
194.0700
Counts vs. Mass-to-Charge (m/z)
d
+EI Scan (rt: 17.119 min) SX_R2_MSTFA_220315.D Subtract
73.0598
75.0300
77.0000
110.0100
147.0600
217.1400
228.1100
184.0700
143.0800
134.0400
Counts vs. Mass-to-Charge (m/z)

e

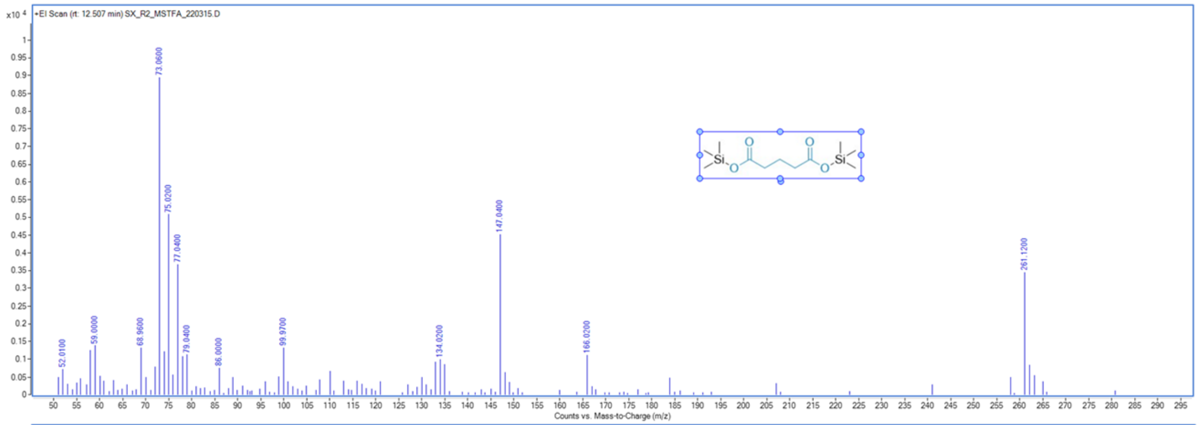

f

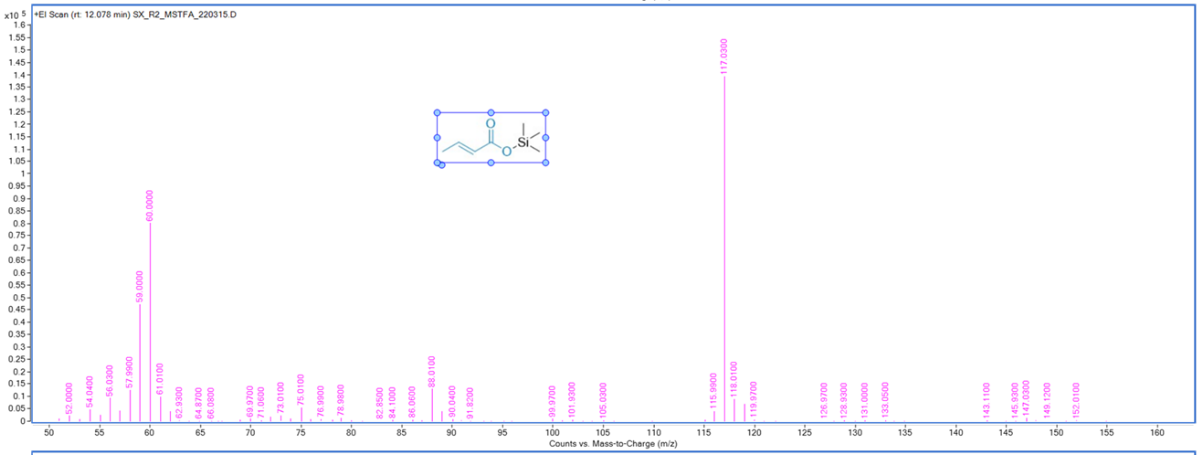

g

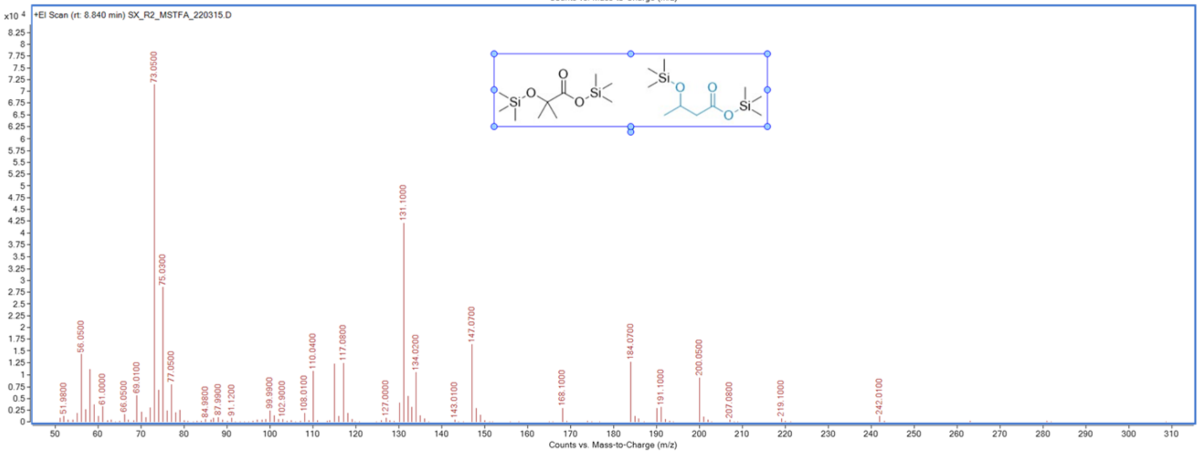

Figure S6. GC-MS analysis of derivatized samples of BES. a) total ion chromatogram, b) 4-hydroxy-benzoic acid derivative ion chromatogram, c) benzoic acid derivative ion chromatogram, d) *(E)*-pent-2-enedioic acid derivative ion chromatogram, e) glutaric acid derivative ion chromatogram, f) *(E)*-but-2-enoic acid derivative ion chromatogram, g) 3-hydroxy-butanic acid derivative ion chromatogram. The sample was taken from long-term BES after the third addition of phenol.

SI-9 Microbial analyses

The acquired reads per sample ranged from 16,590 to 32,485, and a total of 514,000 reads were gathered. Due to rarefaction, 5 (out of 364) ASVs were removed. The rarefaction curves of all samples plateaued (data not shown), showing that samples were sequenced deeply enough to capture the full diversity of bacterial communities. The PERMNOVA showed that the inoculum as well as the microbial communities in BES exposed to different conditions differed significantly ($R^2 = 0.61$, pseudo F-ratio = 8.21, p-value = 0.001).

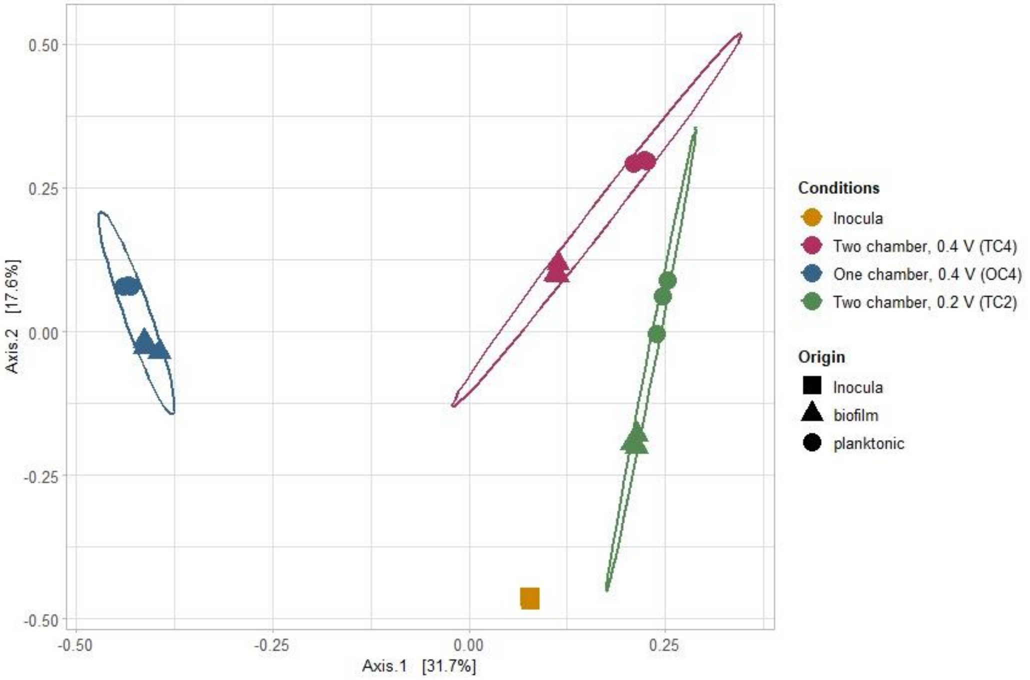

Figure S7. Principal coordinates analysis (PCoA) using square-root transformed Bray-Curtis dissimilarity matrix of microbial samples derived from all BES at the end of each experiment and from the inoculum. The distance-based unconstrained ordination shows that the microbial communities in two chamber systems (TC2 and TC4) were more similar to each other than they were to the ones in the one chamber system (OC4), while all BES samples differed from the inoculum.

References

[1] S. Feisthauer, M. Seidel, P. Bombach, S. Traube, K. Knoller, M. Wange, S. Fachmann and H. H. Richnow. Characterization of the relationship between microbial degradation processes at a hydrocarbon contaminated site using isotopic methods. J Contam Hydrol 133 (2012) 17-29.

[2] S. Dai, B. Korth, L. Schwab, F. Aulenta, C. Vogt and F. Harnisch. Deciphering the fate of sulfate in one- and two-chamber bioelectrochemical systems. Electrochim Acta 408 (2022).

[3] A. Klindworth, E. Pruesse, T. Schweer, J. Peplies, C. Quast, M. Horn and F. O. Glockner. Evaluation of general 16S ribosomal RNA gene PCR primers for classical and next-generation sequencing-based diversity studies. Nucleic Acids Res 41(1) (2013) e1.

[4] Illumina, 16S Metagenomic Sequencing Library Preparation. In (2013).
[5] E. Bolyen, J. R. Rideout, M. R. Dillon, N. A. Bokulich, C. C. Abnet, G. A. Al-Ghalith, H. Alexander, E. J. Alm, M. Arumugam, F. Asnicar, Y. Bai, J. E. Bisanz, K. Bittinger, A. Brejnrod, C. J. Brislawn, C. T. Brown, B. J. Callahan, A. M. Caraballo-Rodríguez, J. Chase, E. K. Cope, R. Da Silva, C. Diener, P. C. Dorrestein, G. M. Douglas, D. M. Durall, C. Duvallet, C. F. Edwardson, M. Ernst, M. Estaki, J. Fouquier, J. M. Gauglitz, S. M. Gibbons, D. L. Gibson, A. Gonzalez, K. Gorlick, J. Guo, B. Hillmann, S. Holmes, H. Holste, C. Huttenhower, G. A. Huttley, S. Janssen, A. K. Jarmusch, L. Jiang, B. D. Kaehler, K. B. Kang, C. R. Keefe, P. Keim, S. T. Kelley, D. Knights, I. Koester, T. Kosciolek, J. Kreps, M. G. I. Langille, J. Lee, R. Ley, Y.-X. Liu, E. Loftfield, C. Lozupone, M. Maher, C. Marotz, B. D. Martin, D. McDonald, L. J. McIver, A. V. Melnik, J. L. Metcalf, S. C. Morgan, J. T. Morton, A. T. Naimey, J. A. Navas-Molina, L. F. Nothias, S. B. Orchanian, T. Pearson, S. L. Peoples, D. Petras, M. L. Preuss, E. Pruesse, L. B. Rasmussen, A. Rivers, M. S. Robeson, P. Rosenthal, N. Segata, M. Shaffer, A. Shiffer, R. Sinha, S. J. Song, J. R. Spear, A. D. Swafford, L. R. Thompson, P. J. Torres, P. Trinh, A. Tripathi, P. J. Turnbaugh, S. Ul-Hasan, J. J. J. van der Hooft, F. Vargas, Y. Vázquez-Baeza, E. Vogtmann, M. von Hippel, W. Walters, Y. Wan, M. Wang, J. Warren, K. C. Weber, C. H. D. Williamson, A. D. Willis, Z. Z. Xu, J. R. Zaneveld, Y. Zhang, Q. Zhu, R. Knight and J. G. Caporaso. Reproducible, interactive, scalable and extensible microbiome data science using QIIME 2. Nat Biotechnol 37(8) (2019) 852-857.
[6] M. Martin. Cutadapt removes adapter sequences from high-throughput sequencing reads. EMBnet.journal 17(1) (2011).
[7] P. Ewels, M. Magnusson, S. Lundin and M. Kaller. MultiQC: summarize analysis results for multiple tools and samples in a single report. Bioinformatics 32(19) (2016) 3047-3048.
[8] B. J. Callahan, P. J. McMurdie, M. J. Rosen, A. W. Han, A. J. A. Johnson and S. P. Holmes. DADA2: High-resolution sample inference from Illumina amplicon data. Nat Methods 13(7) (2016) 581-583.
[9] C. Quast, E. Pruesse, P. Yilmaz, J. Gerken, T. Schweer, P. Yarza, J. Peplies and F. O. Glockner. The SILVA ribosomal RNA gene database project: improved data processing and web-based tools. Nucleic Acids Res 41(Database issue) (2013) D590-596.
[10] P. J. McMurdie and S. Holmes. phyloseq: An R Package for Reproducible Interactive Analysis and Graphics of Microbiome Census Data. PLOS ONE 8(4) (2013) e61217.
[11] M. J. Anderson. Permutational Multivariate Analysis of Variance (PERMANOVA). Wiley StatsRef: Statistics Reference Online (2017) 1-15.
[12] H. Wickham. Reshaping data with the reshape package. J Stat Softw 21(12) (2007) 1-20.
